# Supplementary material for: Are carbon emissions trading and green financial instruments synergistic? -Comprehensive quantitative research based on content analysis
Source: PLoS One. 2024 Mar 7;19(3):e0298601. doi: 10.1371/journal.pone.0298601 (PMC10919601; doi:10.1371/journal.pone.0298601)
Supplement: S1 Data — (ZIP) [file pone.0298601.s001.zip › Data/地级市Z_F工作报告.zip!/║╙▒▒/║Γ╦«╩╨/└┤╫╘╣1⁄2╓┌║┼í╛┬φ┐╦╩2╛▌═°í┐_2019.docx]

各位代表：

　　现在，我代表市人民政府向大会作政府工作报告，请予审议，并请各位市政协委员和列席人员提出意见。

一、2018年工作回顾

　　刚刚过去的一年，是全面贯彻党的十九大精神开局之年，也是我市牢牢把握高质量发展要求、决胜全面建成小康社会取得重大进展的一年。一年来，我们坚持以习近平新时代中国特色社会主义思想为指导，全面落实党中央、国务院和省委、省政府决策部署，在市委正确领导下，坚持稳中求进工作总基调，践行新发展理念，深化供给侧结构性改革，按照“一三五八”总体部署，紧紧依靠全市人民，攻坚克难、真抓实干，统筹推进稳增长、促改革、调结构、惠民生、防风险各项工作，经济社会发展呈现出稳中有进、稳中向好的良好态势。

　　（一）高质量发展取得新成效。预计全市生产总值增长6.9%，固定资产投资增长6.6%，社会消费品零售总额增长9.3%。反映发展质量的指标增幅较高，全部财政收入突破200亿元，增长7%，一般公共预算收入增长11.1%，城乡居民人均可支配收入分别增长9.5%和11%。坚持把科技创新作为高质量发展的第一动力，市本级科技支出由上年的500万元增加到9000万元，着力打造“一谷一港一中心”三大平台，构建“中科院+”“院士+”等协同创新共同体，建成省级产业技术研究院4家、省级技术创新中心26家、省级重点实验室1家、院士工作站17家、各级科技企业孵化器14家、众创空间34家、省级以上星创天地38家，新认定高新技术企业113家、科技型中小企业850家。坚持把项目建设作为高质量发展的重要支撑，139个省、市重点项目完成投资451.5亿元，为年计划的148.2%，武强和平铝业高端铝型材加工、桃城区海博呈盛数字金融智造基地等97个项目开工建设，衡水高新区中广核生物天然气工程、冀州“果维健”大健康产业园等33个项目顺利投产，形成了一批新的经济增长点。

　　（二）转型升级迈出新步伐。实施“产业转型升级年”攻坚行动，出台支持工业设计发展的十条措施，对1000余家规模以上工业企业一企一策制定转型升级路线图，培养引进万名企业科技及管理骨干，列入省技改项目数量和争取资金数额全省领先。高质量绿色农业扎实推进，以饶阳为核心区的衡水国家农业科技园区成功获批，在全省率先实现省级现代农业园区县域全覆盖，粮食总产80亿斤，注册家庭农场、农民合作社数量、土地流转率均居全省首位。现代服务业加快发展，电商企业增至3.8万家，京东中国特产衡水馆上线运行，武强、阜城列为国家级电子商务进农村综合示范县，市第二届旅发大会成功举办，全年旅游业总收入比上年增长20.5%。

　　（三）城乡面貌呈现新变化。实施“3个10”市政重点工程，植物园、野生动物园、孔颖达公园等建成开园，奥体中心体育场、市垃圾综合处理厂顺利建成，人民路东延、大庆路东延主体完工，市文化艺术中心建成投用，结束了我市没有大剧院、图书馆和公立博物馆的历史。推进城市精细化管理，取缔马路市场11处，新改建便民市场15个，改造提升市区路口20个，建成公厕57座，顺利通过省级卫生城市验收，国家园林城市、国家森林城市等创建工作成效显著。实施县城建设攻坚项目259个，完成投资174.2亿元，枣强县、安平县和武邑县清凉店镇入列省级新型城镇化与城乡统筹示范区试点，8个乡撤乡建镇。狠抓农村人居环境整治，全市4992个村全部完成积存垃圾清理，改造农村厕所3.5万个，启动乡村振兴示范区13个，“三区同建”省级示范点达到74个。全省县城建设攻坚行动现场观摩会、全省实施乡村振兴战略暨农村人居环境整治工作现场会在我市召开。

　　（四）改革开放释放新活力。全力打造“放管服”改革衡水升级版，推行“双随机一公开”监管，加强市县行政审批局标准化建设，推出“一窗受理”“一照通”和电子招投标平台等系列改革举措，市本级“最多跑一次”事项达到380项，占全部政务服务事项的64.3%，全市市场主体达到30.6万户，增长11.7%。省级开发区人事薪酬制度改革扎实推进。县级融媒体中心全部挂牌成立，武强融媒体改革得到中央领导肯定。第二批11个乡镇体制改革启动实施。“手机扫码乘公交”等微改革微创新项目受到群众欢迎。外贸出口逆势上扬，出口总值增长8%，实际利用外资增长12.6%。成功举办衡水京津冀“双协同”创新论坛、河北银行业支持衡水经济发展座谈会等活动，赴外地招商1192批次，与京津对接合作项目634个，引进雄安新区转移升级企业100余家，与中核工等30余家战略投资者签署框架协议。瑞丰动力、养元饮品成功上市。

　　（五）生态建设得到新加强。扎实做好中央、省环保督察“回头看”问题整改，大力开展污染治理八大专项行动，在全省率先出台生态环境违法“黑名单”管理办法，排查整治“散乱污”企业508家，完成“气代煤”“电代煤”27.07万户，建筑工地全部安装PM10在线监测设备和视频监控设施，公交车免费乘坐期延至6个月，150部新能源公交车上线运营，空气质量稳定退出全国74个城市后10名、169个城市后20名，优良天气增加天数位居全省第一。地下水超采综合治理扎实推进，全年引蓄外来水8.6亿立方米。河湖水质普遍提升，我市首部地方性法规——《衡水湖水质保护条例》将于3月1日起施行。全年造林39.2万亩，森林覆盖率达到32%。

　　（六）人民生活实现新改善。举全市之力坚决打好脱贫攻坚战，武邑、饶阳、阜城、枣强脱贫摘帽，武强、故城通过省脱贫退出验收，152个贫困村出列，2.5万贫困人口脱贫，阜城被评为国家健康扶贫工程先进县。在全省率先开展民生实事项目人大代表票决制，在入学、就医、出行、文化、健身等方面为群众办成了一批实事好事。城镇新增就业4.58万人，登记失业率3.37%。完成棚户区改造6567套，改造农村危房5052户。主城区52个住宅小区配建幼儿园收由政府管理，新改扩建中小学8所，“入园难”“入园贵”和中小学班容量过大问题得到缓解。组建全省首个紧密型城市医疗集团，成立21个医联体，基层医疗机构就诊率大幅增长。市中医院晋升三级甲等医院，市二院晋升三级综合医院。成功举办2018衡水湖国际马拉松赛、衡水航空运动嘉年华暨全国动力伞精英赛、中华民族大赛马等活动，群众文娱生活更加丰富多彩，精神文明创建深入开展。社会治理水平不断提升，退役军人服务管理工作得到加强，房地产遗留问题中一大批征收难、入住难、办证难问题得以解决。平安衡水建设取得扎实成效，扫黑除恶专项斗争深入推进，安全生产和食品药品安全形势持续向好，社会大局保持和谐稳定。

　　政府自身建设进一步加强，坚决落实全面从严治党战略部署，大力推进政府系统党的建设。认真做好中央和省委巡视反馈意见整改，坚持以大督查促进重点工作落实，严格落实中央八项规定精神及其实施细则，建立健全各项制度，政府公信力不断提升。加强法治政府建设，认真学习宣传贯彻《宪法》，自觉接受人大法律监督、工作监督和政协民主监督，全年办理人大代表建议和政协提案573件，办结率、满意率均为100%。国防动员和“双拥”共建工作深入开展，民族宗教、新闻出版广电、外事侨务、人民防空、史志档案、防灾减灾、援藏援疆、妇女儿童、老龄、残疾人等工作都取得了新的成绩。

　　各位代表，过去一年，在外部环境复杂多变、各方面任务异常繁重的情况下，取得这样的成绩确实不易。这是习近平新时代中国特色社会主义思想科学引领的结果，是省委、省政府和市委正确领导的结果，是全市人民齐心协力、奋力拼搏的结果，是社会各界鼎力支持的结果。在此，我代表市人民政府，向全市人民，向人大代表、政协委员，向各民主党派、工商联、无党派人士和人民团体，向驻衡部队、武警官兵、政法干警和中省直驻衡各单位，向所有关心支持衡水改革发展的各界朋友，致以崇高的敬意和衷心的感谢！

　　各位代表，我们也清醒地看到，全市发展仍面临不少困难和问题。发展不充分不平衡仍是当前的主要矛盾；实体经济发展困难较多，经济下行的压力还比较大；产业结构调整还处于爬坡过坎阶段，科技创新能力还不够强；改革开放需持续深化，发展的动力活力还有待进一步增强；科技、教育、医疗等方面的投入不足，民生领域还有不少短板；不安定不稳定因素仍然存在，防风险守底线的任务还比较繁重等。对这些问题，我们一定高度重视，采取有力措施，认真加以解决。

二、2019年工作总体要求和主要目标

　　今年是新中国成立70周年，是全面建成小康社会、实现第一个百年奋斗目标的关键之年，做好今年工作至关重要。习近平总书记对河北工作的重要指示批示精神，为我们贯彻新发展理念、推动高质量发展、夺取决胜全面建成小康社会新胜利指明了方向、提供了行动指南。省委、省政府系列决策部署，为我们做好工作进一步明确了目标任务。近年来，在市委正确领导下，全市发展形成了稳中向好、增速领先、增比进位的良好态势，后发优势、区位优势、生态优势、营商环境优势进一步彰显，广大干部群众盼发展、谋发展的愿望迫切、干劲十足，为加快发展提供了强大支撑。我们一定要坚定信心、乘势而上，不断开创新时代衡水高质量发展新局面。

　　今年政府工作的总体要求是：坚持以习近平新时代中国特色社会主义思想为指导，全面贯彻党的十九大和十九届二中、三中全会精神，认真落实中央经济工作会议、省委九届八次全会和市委四届四次全会精神，牢牢守住稳定、发展、生态三条底线，坚定走好创新引领、科技支撑、绿色发展之路，深入落实“一三五+”总体部署，扎实推进“八大工程”，加快建设现代化经济体系，继续打好三大攻坚战，深入开展“双创双服”活动，深化改革、扩大开放，普惠民生、拼搏奋进，保持经济持续健康发展和社会大局稳定，为全面建成小康社会收官奠定决定性基础，以优异成绩庆祝中华人民共和国成立70周年。

　　主要预期目标是：全市生产总值增长7%左右，一般公共预算收入增长7%，规模以上工业增加值增长5%，高新技术产业增加值增长12%，固定资产投资增长6.5%以上，社会消费品零售总额增长10%，进出口总值增长5%；城镇登记失业率控制在4.5%以内；城乡居民人均可支配收入分别增长8.5%和9.5%；居民消费价格涨幅控制在3%左右；万元生产总值能耗、PM2.5平均浓度降幅及化学需氧量、二氧化硫、氨氮、氮氧化物减排完成省下达目标任务。这些指标的设定，充分考虑了各方面支撑条件和需要，兼顾了总量增长和结构优化，与“十三五”规划目标相衔接，经过努力是可以实现的。实际工作中，争取发展得更好更快些。

　　完成上述目标任务，必须坚持以习近平新时代中国特色社会主义思想为指导，切实把思想、认识和行动统一到习近平总书记对河北工作的重要指示批示精神上来，自觉践行新发展理念，时时处处事事对标对表，确保各项工作沿着正确方向推进。必须坚持稳中求进工作总基调，正确处理“稳”和“进”的辩证统一关系，既扎实做好稳就业、稳金融、稳外贸、稳外资、稳投资、稳预期工作，又着力在转型升级、优化结构、提高质量上奋发作为，全力推动高质量发展。必须坚持以供给侧结构性改革为主线，贯彻“巩固、增强、提升、畅通”八字方针，实质性降低企业各类营商成本，补齐基础设施和公共服务短板，提升产业发展水平，毫不动摇支持民营经济高质量发展，不断增强我市实体经济竞争力，夯实富民强市的物质基础。必须坚持“一三五+”总体部署，把建设京津冀发展环境高地作为赢得区域竞争优势的先手棋，把迈进高质量时代、智慧城市时代、千亿产业时代作为跨越赶超的战略抓手，把打造实力衡水、创新衡水、生态衡水、文化衡水、幸福衡水作为我们的梦想和追求，紧盯目标，做实载体，一年接着一年干，一张蓝图绘到底。必须坚持以改革开放创新为动力，在做实综合配套改革上下功夫，在推动高水平开放上下功夫，在引入高端要素、提升创业创新水平上下功夫，在持续优化营商环境上下功夫，积极创建全国质量强市示范城市，为我市经济社会发展注入强大动力。必须牢固树立以人民为中心的发展思想，坚持为了群众、相信群众、依靠群众，从基层群众中汲取智慧和力量，尽力而为、量力而行，按照人民意愿办好民生实事，不断增加群众的获得感、幸福感、安全感。

三、2019年重点工作任务

　　今年，市委、市政府谋划了产业提档升级、非贫困县非贫困村脱贫攻坚、生态文明创建、城乡品质塑造、乡镇强身健体、智慧医疗体系培育、教育规范提升、县城交通互联互通等“八大工程”。这“八大工程”既是当前推动高质量发展、保障和改善民生的当务之急，也是着眼可持续发展打基础、利长远的工作。我们要以“八大工程”为全市工作的总抓手和突破口，逐项制定方案，集中力量推进，带动和活跃发展全局。以此为基础，统筹抓好七个方面工作:

　　（一）千方百计扩大有效投资，增强经济发展后劲。树牢项目不可替代意识，坚持把项目作为第一抓手，努力以高质量投资拉动经济持续健康发展。

　　着力推进“双基”工程建设。基础设施和基本公共服务项目体量大、带动能力强，是稳投资、稳增长的“底盘”。抢抓当前国家增加基础设施投资规模、建设成本较低的机遇，在交通、水利、能源、市政、生态环保等领域，加紧谋划实施一批重大项目，力促雄商高铁、石衡高速、衡水到雄安一级公路、郑昔线冀州段尽早开工，加快推进石衡沧港城际铁路、邯港高速、京德高速前期，抓好高铁新城、滏阳河文化带建设和老盐河片区改造，加快衡水学院新院区、哈院新院区、衡水高新区医院建设。

　　着力推进产业项目建设。坚持把产业项目作为发展实体经济、优化投资结构的重中之重，实施“112”工程。抓好100个重点开工在建项目，加快推进正大肉鸡、东旭环保产业园等重大项目。推进100个重点前期项目，力争海伟聚丙烯二期、高科智能温控传导系统等项目年内开工。围绕培育主导产业，谋划重点产业项目200个，抓紧完善前期手续，充实项目库，形成谋划一批、储备一批、建设一批、投产一批的滚动发展格局。着力提升园区能级和水平。大力开展园区对标提升、“二次创业”活动，当好改革开放急先锋、招商引资主力军，树立高质量发展的新标杆。支持衡水高新区打造千亿产业园，力争年内晋升国家级高新区。支持滨湖新区建设国家5A级景区，着力在发展教育实训、研发设计、文体旅游、健康休闲等方面求突破。支持县市区园区依托骨干企业，引入高端要素，完善配套功能，坚定不移朝着“专、精、新、特”方向发展。全市园区规模以上工业主营业务收入增速达到10%以上。

　　着力完善项目推进机制。强化重点项目领导包联和全程帮办，及时协调解决实际问题。对新建项目，严格投资强度、容积率、回报率、开工率要求，土地指标随着税收走、随着进度走，对工作实、推进快、效益好的地方重点保障。实行周督月调制度，集中开工、观摩拉练由上年的2次增加到3次，以产业项目及首期投资为重点，突出项目质量、效益导向，完善考评奖惩办法，形成比学赶超上大项目、好项目的浓厚氛围。

　　（二）推进产业转型升级，夯实高质量发展的基石。坚持主动调、加快转，聚焦“3+2”产业发展方向，推进产业重构、布局重廓、企业重组，努力向产业链、技术链、创新链、价值链中高端迈进。

　　改造提升传统产业。深入开展“千企转型”行动，推动丝网、玻璃钢等10大特色产业优化升级，对标行业先进水平，积极寻求战略合作，全面转向创新驱动、绿色发展、现代管理。加大技改投入，市本级设立不少于1500万元技改专项资金，全市争取实施省、市重点技改项目100个以上。加强工业设计，建成启用市级工业设计创新中心和2个县级中心，实施工业设计示范项目10个以上。加快“两化”融合，建成数字车间5个以上，完成企业“上云”100家以上。出台激励政策，支持“个转企”“小升规”，今年新增规模以上工业企业70家、挂牌上市企业10家以上。

　　加快培育战略性新兴产业。在完善新兴产业链上下功夫，聚焦高端装备制造、新材料、生物医药与医疗器械、新一代信息技术等重点领域，研究制定专项规划，明确主攻方向，加大产业链缺失环节招商力度，在补齐增强上取得新突破。在未来产业布局上下功夫，谋划发展服务机器人和数字产业，突出抓好数字金融和信息安全基地、九次方衡水京南大数据清洗基地等项目建设，与中科院合作建设超级计算中心，打造高新技术产业局部强势。在推进军民融合发展上下功夫，谋划建设军民融合产业基地1-2个，培育示范企业3-5家。

　　推进服务业提档升级。加快制造业与服务业深度融合，发展物流、教育、文化、旅游、康养等领域新兴业态，推动服务业总量增长、结构优化、效率提升。抓好京东衡水电商产业集聚中心项目，鼓励县市区依托第三方电商平台开设地方特色馆，谋划建设中铁物流园，推动安平丝网聚成物流园改造升级，加快爱琴海国际购物中心等城市综合体建设。大力发展全域旅游，抓好中国马城、香料小镇等重点项目建设，完善旅游自助服务设施，推出衡水“两日游”精品线路，举办市第三届旅发大会。立足衡水教育优势，谋划发展与教育相关的产业，更好满足各级各类教育对衣食住行的需求，更好开展教育教学个性化培训服务，同时积极发展教学器材和装备研发制造等产业。

　　做强县域特色经济。县域特色经济是衡水的靓丽名片，是高质量发展的重要基础。引导县市区在全国、全省大格局中找准位置，强力对标赶超，支持景县、深州、安平树立更高标准，明确进入全省30强时间表、路线图，在全市实现率先发展。落实市委做大做强特色乡镇的部署，依托现有产业，选择基础较好、优势明显的乡镇重点培育，用3年时间集中打造30个产业聚集型、文化旅游型、现代农业型、康泰养生型、商贸物流型特色小镇，形成县域经济新的增长极。

　　我市县域经济的主体是民营经济。民营兴、县域兴，民营活、衡水活。我们将认真贯彻习近平总书记关于支持民营经济发展的重要论述精神，全面落实中央、省出台的一系列支持政策，加快构建亲清新型政商关系。围绕解决民营企业融资难、融资贵问题，引导金融机构强化与实体经济共生共荣的理念，鼓励银行提高存贷比，增加中长期贷款，对优质骨干企业区别对待，确保不抽贷、不停贷、不断贷。实施特色产业迭代升级“五个一”工程：建立一个公共平台，支持每个特色产业深度对接国内外知名高校、科研院所，建立产业研究院或行业标准检验检测中心，增强产业发展的科技支撑能力；成立一个行业协会或商会，实现“抱团出海”“抱团闯市场”，防止恶性竞争；培育一家上市企业，用好上海科创板设立等机遇，争取每年实现一家企业上市；推出一个标准或知名品牌，支持企业创制国标、省标和行业标准，培育做强特色品牌；开展一次专业培训，通过早餐会等方式促进企业家交流，提升企业管理水平。

　　（三）实施乡村振兴战略，加快推进农业农村现代化。坚持农业农村优先发展，积极探索具有衡水特色的乡村振兴之路，推动农业增效、农村发展、农民增收。

　　加快发展现代农业。深化农业供给侧结构性改革，大力发展科技农业、绿色农业、品牌农业和质量农业，在巩固粮食生产能力前提下，扎实推进“一减四增”，重点实施“5116”工程，推动农业提质增效。积极发展现代都市型农业，在主城区、衡水湖周边和有条件的城镇周边，布局一批集育种科研、观赏体验、休闲娱乐于一体的田园综合体，重点抓好桃城区“绿港”和饶阳贵澳等园区建设，支持安平生猪产业园创建国家现代农业产业园。加快培育农业特色产业，按照“一县一业一品一特色”思路，因地制宜发展规模农业，深化与中国农科院、省农科院合作，推进科技成果转移转化，提升农业竞争力和质量效益。

　　全面改善农村人居环境。抓好农村垃圾污水治理、厕所革命和村容村貌提升，健全农村生活垃圾收运系统，促进城乡生活垃圾处理一体化，年内完成农村厕所改造20万个。结合“空心村”治理和空闲农宅利用，建设一批小游园、小菜园等“微绿地”。用足用好城乡建设用地增减挂钩和耕地占补平衡政策，加快推进“三区同建”，今年每个县市区集中打造1个重点片区，启动3个示范点。加强农村基础设施建设，搞好村内道路硬化、绿化、亮化，持续抓好“四好农村路”建设，加快农村客运班线公交化改造，实施江水城乡供水一体工程，增加覆盖人口60万以上。

　　多渠道促进农民增收。培育新型农民，组织开展实用技能培训，对有创业意愿者提供创业资金扶持，发展特色种养、农家乐、特色手艺等，让更多群众找到适合自己的致富门路。发展劳务经济，完善与输入地劳务对接机制，打造一批叫得响的衡水劳务品牌，切实保障外出务工人员权益。改进土地流转，注重对接农研团队、金融资本、销售网络，发挥龙头企业带动作用，强化利益联结机制，发展一批集科研、生产、加工、销售于一体的现代农业联合体，农业产业化经营率保持在69%以上。

　　（四）实施城乡品质塑造工程，展现美丽衡水新风貌。加强城乡规划建设管理，完善功能布局，持续提升城乡品质，加快建设生态宜居滨湖园林城市。

　　坚持规划引领。优化全市空间开发模式，完善“五级两规一导则”，构建“多规合一”空间规划体系，推进经济社会发展与城乡建设、产业、土地利用、生态环境保护等规划有机衔接，实现全市“一张图”管控。加快编制县域空间规划，各县市在做好县城规划的同时，增强全域规划观念，统筹生产、生活、生态三大空间布局，科学规划需要保留和撤并的村庄，以村庄撤并的“减法”换取新型城镇化和乡村振兴的“乘法”。

　　做强做美中心城区。让城市经济强起来，在优化中心城区产业布局基础上，高标准谋划建设中央商务区，发展楼宇经济，下力量引进国内外知名企业在衡设立区域总部，积极引进名品名店和金融、科技、结算中心等高端企业，增强城市经济实力。让中心城区美起来，对标对表国内外先进城市标准，研究制定生态城市、宜居城市、园林城市3个实施方案，开展达标提标专项行动，今年重点推进前进街、人民路等市区干道改造提升工程，加快建设滏东公园、滏阳河市区段带状体育公园和星河湾等一批街头游园。让城市管理精细起来，扎实开展城市管理治乱攻坚工程，整治不合规划的沿街门店、机动车非机动车乱停乱放、马路市场和占道经营，以及随意设置的广告和门店牌匾；推动智慧衡水建设，年内实施地理信息库、“城市通”市民融合平台等重点项目。加大对冀州区融入主城区支持力度，在政策和基础设施建设上予以倾斜。发挥多城同创的带动作用，高标准做好各项工作，确保每个指标都有把握稳定达标、通过验收。发挥好重大活动的带动作用，办好2019衡水湖国际马拉松赛、2019国际智力运动联盟世界大师锦标赛（衡水）、中国（衡水）纪录片节暨衡水湖国际纪实影像放映周、市第四届运动会，促进城乡基础设施和公共服务水平显著提高。

　　提升城镇吸引力和承载力。完善县城公共服务设施，建设公园、绿地和市民休闲带，改造城镇水系，加快建成一批新地标、打造一批新景观、形成一批新亮点，每个县城至少有1个四星级以上精品公园。围绕建设北方最洁净城镇群的目标，推广先进县市做法，统筹通信线缆入地、沿街立面改造等工作，塑造疏朗有致的天际线，提高城镇的洁净度、美誉度。推进“海绵城市”建设，实施排水管网雨污分流改造。支持枣强创建国家园林县城。实施乡镇强身健体工程，扎实做好规划编制、功能完善、面貌提升等工作，支持每个乡镇发展1个特色主导产业。

　　（五）深入推进改革开放创新，激活经济发展活力。赋予综合配套改革试验市新内涵，持续深化各领域改革，扩大高水平对内对外开放，加快推进科技创新，为经济社会发展注入新的动力和活力。

　　坚定不移深化市场化改革。围绕解决项目落地难问题，深化“放管服”改革，完成企业开办全程网上办、行政许可审批服务标准化两个国家试点改革任务，对产业项目探索试行承诺制、“标准地”改革，在省级以上开发区试行“三零”审批，对政府投资的“双基”项目，借鉴“一会三函”做法实施行政审批流程再造，构建审批事项最少、办事流程最优、办结时间最短的行政审批新模式。围绕补上基本公共服务短板，加快教育文化卫生领域改革，促进教育全面、规范、有序发展，推进医疗、医保、医药、医价联动改革，着力解决群众看病难、看病贵问题。围绕解决科技创新能力不足、高端人才紧缺问题，深化科技体制改革，激发科技人才的创新动力，结合产业发展需求，有针对性地吸引国内外知名专家、团队和优秀高校毕业生来衡创业兴业，用好双创专项金融债券和孵化专项债券。围绕盘活农村资产资源，深化农业农村改革，探索推动“三块地”改革，推进农村承包地和宅基地两个“三权分置”，搞活农村金融。同时，完成党政机关机构改革，深化国资国企改革，稳妥推进经营性国有资产集中统一监管改革，进一步深化金融财税体制等改革。

　　坚定不移扩大高水平开放。在推动京津冀协同发展中扩大开放，精准承接北京非首都功能疏解，争取协同发展“微中心”落户衡水，主动对接服务雄安新区，搭建与之配套的产业孵化基地、雄衡合作产业园等合作平台，推进深州北京绿色家具产业园、阜城中华老字号食品产业园和故城雄县产业融合生态城建设。在开展精准招商中扩大开放，出台加强招商引资工作若干政策措施，着眼做大做强主导产业，锁定目标科技团队、目标龙头企业、目标销售网络，开展“敲门招商”，提高招商实效。用好省香港招商活动、廊坊5·18经洽会等重要平台，举办衡水京津产业转移大型招商会。紧盯改革开放前沿地区，坚持引项目、引资金和学制度、学规则并重，深化合作共赢。在推动“三外联动”中扩大开放，抓好安平丝网、故城裘皮、大营裘皮3个国家级出口基地和省级精细化工出口基地建设，谋划建设综合保税物流中心和海外仓，巩固外贸大市地位。引导出口企业和相关企业放宽眼界，携手发展，以外贸带动外资、外经，实现“三外”互促并进。

　　坚定不移实施创新驱动。多渠道增加科技投入，全社会研发支出比上年增长10%。发挥创新平台作用，依托“一谷一港一中心”，推动与中科院、中国农科院等战略合作协议落地落实，新建一批重点实验室、技术创新中心、工程研究中心和企业技术中心。加强厅市合作，全力推进一批会商项目，集成科技资源，吸引先进科技成果落户衡水。培育壮大创新主体，继续实施科技倍增计划，年内新认定高新技术企业118家、科技型中小企业750家。加快科技成果转化，统筹科创资源，加强对重点企业会诊，嫁接孵化科技成果100项以上。实施人才强市战略，年内至少引进2个高层次团队，新增2家院士工作站。

　　坚定不移打造京津冀发展环境高地。在加强政企有效对接上增措施，开通政企联系直通车，建立涉企政策信息集中公开制度和发布推送制度，定期组织召开企业家恳谈会，成立地市级以上驻外地商（协）会10家以上，进一步密切与国外华商会、同乡会的招商联系。在降低企业经营成本上出实招，全面落实上级减税降费政策，对地方权限内有关税费政策，在国家规定的幅度内降到最低水平，推动政府定价涉企经营服务性收费项目只减不增、标准只降不升。在更好服务企业上用真力，鼓励公职人员与企业家交朋友，实行企业首次一般性过错提醒免罚，规范和减少涉企检查，推行环保差别化执法。在“双创双服”上求实效，围绕“创新、强企、解难、惠民”，及时协调解决实际问题，精准服务高质量发展。

　　（六）打好“三大攻坚战”，补齐全面建成小康社会短板。坚持目标导向，针对突出问题，全力攻坚突破，力争取得明显成效。

　　打好防范化解重大风险攻坚战。落实国家总体安全观，增强忧患意识，切实防范各类风险挑战。防范和化解区域性金融风险，深入开展“金融港”护航计划和拆圈解链专项行动，健全“府院联动”机制，严厉打击非法集资和恶意逃废债行为。坚决遏制政府隐性债务增量，逐步消化政府债务存量。严格落实安全生产责任制，深入开展安全生产大排查大整治大执法攻坚行动，坚决防止重特大事故发生。加大食品药品抽检力度，确保食品药品安全。加大“非洲猪瘟”等动物疫情监测防控力度，确保畜牧业安全稳定发展。认真做好社会矛盾排查化解，妥善处置信访多发领域问题。完善社会治安防控体系，深化扫黑除恶专项斗争，加快建设平安衡水、和谐衡水。

　　打好精准脱贫攻坚战。下大力巩固脱贫成果，健全扶贫长效机制，对脱贫人口完善后续扶持措施，脱贫不脱帮扶、脱贫不脱政策、脱贫不脱项目、脱贫不脱监管。对非建档立卡低保户、低收入边缘户和已脱贫但收入不稳定、持续增收能力不强的脱贫户，纳入防贫监测范围，有针对性地扶持救助。对符合条件的新致贫户，及时按规定程序纳入建档立卡范围，落实增收项目和“三保障”扶持政策。下大力推进非贫困县非贫困村脱贫攻坚工程，以促进贫困群众稳定增收为核心，通过加大资金投入、改善基础设施、推进产业就业等措施，加快脱贫奔小康步伐。今年力争60个贫困村出列、1.6万贫困人口脱贫，为2020年全面打赢脱贫攻坚战奠定坚实基础。

　　打好污染防治攻坚战。坚决打赢蓝天保卫战，坚持科学治霾、铁腕治霾、精准治霾，开展“散乱污”企业动态“清零”行动，扎实推进清洁取暖，深入开展柴油货车污染、扬尘污染、挥发性有机物污染治理，有效应对重污染天气，确保空气质量持续改善。坚决打赢碧水保卫战，加强城镇污水专项治理，年内主城区和县城污水处理率分别达到95%和90%，城市建成区内黑臭水体和农村纳污坑塘实现“双清零”，巩固提升农村饮水安全，实行最严格的水资源管理制度。坚决打赢土壤污染治理攻坚战，加强涉重金属行业污染防控，推进污染地块土壤环境调查评估。坚决打赢生态修复攻坚战，强力推进地下水超采综合治理，搞好衡水湖及周边地区生态保护与修复，确保水环境质量持续稳定改善，大力度开展环湖、环中心城区、县城、镇村林带建设及沿交通干线廊道国土绿化，全年新造林25万亩。

　　（七）着眼民急民需民盼，努力保障和改善民生。今年，我们在广泛征求民意基础上，以“使用”为标准，谋划了25项备选民生实事，待这次大会票决后，逐项制定实施方案，确保高标准高质量完成。

　　精心做好社会保障工作。大力促进就业创业，突出抓好高校毕业生、农民工、退役军人、城镇失业人员等群体就业工作，城镇新增就业4.5万人。强化住房保障，今年棚户区改造开工3495套、基本建成2930套，改造老旧小区126个、农村危房1237户。推进低保、特困对象精准认定和指导标准动态调整，完善老年人家庭赡养和扶养、社会救助、社会福利等政策，探索集中供养新模式，保障困难群众基本生活，织密织牢兜底民生保障网。

　　巩固扩大教育优势。坚持立德树人，学前教育抓普惠普及，义务教育抓优质均衡，高中教育抓改革发展，职业教育抓产教融合，高等教育抓内涵提升，培养德智体美劳全面发展的社会主义建设者和接班人，把衡水教育名片擦得更亮。深入开展消除中小学大班额专项行动，新改扩建中小学、幼儿园69所。在主城区所有公办小学全面推行下午放学后免费托管服务，开展公办中小学营养午餐试点，鼓励有条件的县市积极试行。推进衡水中学扩建、衡水交通职教园建设，启动滏阳中学新建前期工作。

　　繁荣发展文化事业。积极培育和践行社会主义核心价值观，实施中华优秀传统文化传承发展工程，完善现代公共文化服务体系，加快市图书馆全面对外开放，推动文化院馆更好惠及大众，推进市博物馆展陈工作，落实对民营博物馆的扶持政策。继续开展“政府采购、送戏下乡”、高雅文化进衡水等文化惠民活动，举办庆祝建国70周年系列文化活动。在国家博物馆举办“文华衡水”展，办好2019中国衡水董仲舒与儒家思想国际研讨会。开展大运河文物保护工程和大运河展示项目建设。

　　实施智慧医疗体系培育工程。建设市、县两级远程医疗综合管理和运营监管平台，推进远程医疗服务覆盖所有医疗联合体和县级医院。加快推进城市医疗集团建设，健全分级诊疗体系，优化医疗资源配置，推进京衡中医药“名片”工程落地落实，支持康复医疗器械产业和大健康产业加快发展。

　　加强国防教育和后备力量建设，强化退役军人服务保障，维护军人军属合法权益，让军人成为全社会尊崇的职业。充分发挥工会、共青团、妇联等人民团体桥梁纽带作用。扎实做好民族宗教、新闻出版广电、外事侨务、人民防空、史志档案、气象地震、援藏援疆、妇女儿童、老龄、残疾人等工作。

四、努力建设人民满意政府

　　各位代表，高质量发展重任在肩，决胜全面小康时不我待。我们一定不忘初心、牢记使命，进一步加强政府自身建设，努力提升履职能力和服务水平，切实做到对人民负责、让人民满意，不断开创政府工作新局面。

　　（一）旗帜鲜明讲政治。高举习近平新时代中国特色社会主义思想伟大旗帜，深入贯彻落实总书记对河北工作的重要指示批示精神，树牢“四个意识”、坚定“四个自信”、落实“两个维护”，始终在思想上政治上行动上同以习近平同志为核心的党中央保持高度一致，确保中央和省各项决策部署在衡水落地见效。

　　（二）深入推进依法行政。坚决维护宪法法律权威，依法全面履行政府职能。自觉接受人大法律监督、工作监督和政协民主监督，认真办好人大代表建议和政协提案，主动接受社会监督和舆论监督。切实推进政务公开和政府信息公开，加强行政规范性文件合法性审核，健全政府法律顾问制度，严格规范行政执法行为。

　　（三）全面提高服务效能。适应贯彻新发展理念、推动高质量发展要求，着力提高各级干部的执行力、创新力，坚决克服庸懒散现象。强化公仆意识，坚持问计于民、问需于民，确保政府决策顺民心、合民意。认真落实政府服务承诺，做到言必信、行必果，踏踏实实把衡水的事情做好，以实干取信于民，以实绩造福于民。

　　（四）切实加强廉政建设。认真落实全面从严治党主体责任和“一岗双责”，全面推进党的政治建设、思想建设、组织建设、作风建设、纪律建设，强力推进中央、省委巡视反馈意见整改落实。严格执行中央八项规定精神及其实施细则，驰而不息反“四风”、树新风，坚决反对官僚主义、形式主义，深入推进党风廉政建设和反腐败斗争，持之以恒正风肃纪，努力做到为民、务实、清廉。

　　各位代表，奋斗成就伟业，实干赢得未来。让我们更加紧密地团结在以习近平同志为核心的党中央周围，在省委、省政府和市委正确领导下，坚定信心，接续奋斗，开拓创新，为新时代全面建设经济强市、美丽衡水作出新的更大贡献，以优异成绩庆祝中华人民共和国成立70周年！
